# Mefenamic acid-induced bilateral transient myopia, secondary angle closure glaucoma and choroidal detachment

**DOI:** 10.4103/0301-4738.55066

**Published:** 2009

**Authors:** Parag Vishwakarma, Ganesh V Raman, P Sathyan

**Affiliations:** Glaucoma Services, Aravind Eye Hospital, Avinashi Road, Coimbatore-641014, India

**Keywords:** Mefenamic acid, secondary angle closure glaucoma

## Abstract

Drug-induced secondary angle closure is quite common and in the majority of cases simply stopping the medication leads to rapid reversal of the condition and resolution of glaucoma. We describe here a patient who presented with secondary angle closure glaucoma and myopia following mefenamic acid ingestion which was managed successfully by stopping the medication, symptomatic treatment and reassurance.

Transient myopia with bilateral secondary angle closure glaucoma is a well-documented complication of several drugs such as topiramate,[1] sulfanilamides,[2,3] hydrochlorthiazide,[4] and acetazolamide.[5] Although nonsteroidal anti-inflammatory drugs (NSAIDs)[6] have been known to cause transient myopia they have so far not been reported with secondary angle closure glaucoma. When such an episode occurs, rapid resolution is effected by stopping the medication and observation as in the majority of patients the symptoms are transient. We report a case of secondary angle closure glaucoma following ingestion of mefenamic acid.

## Case Report

A 30-year-old male patient presented to us with diminution of vision, associated with pain, and colored haloes in both eyes for two days. He had been suffering from headache for one week before presentation for which he was prescribed meftal tablet 500 mg (mefenamic acid, Blue Cross Laboratories Ltd, India) by a local general practioner. He gave a history of having taken one tablet each four days apart following which his present symptoms commenced. He consulted a local ophthalmologist in his hometown who measured the intraocular pressure (IOP) (by Schiotz tonometer – unrecordably high) and prescribed eye drops timolol maleate 0.5% twice daily, pilocarpine 2% thrice daily, chloramphenicol-dexamethasone combination four times daily and tablet acetazolamide 250 mg thrice daily. The patient used the prescribed medication for one day before he presented to us.

**Figure 1:**
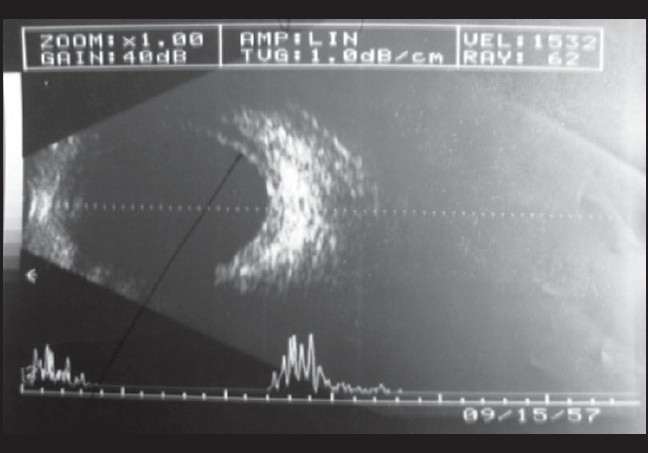
Right eye USG B-scan: choroidal detachment

**Figure 2:**
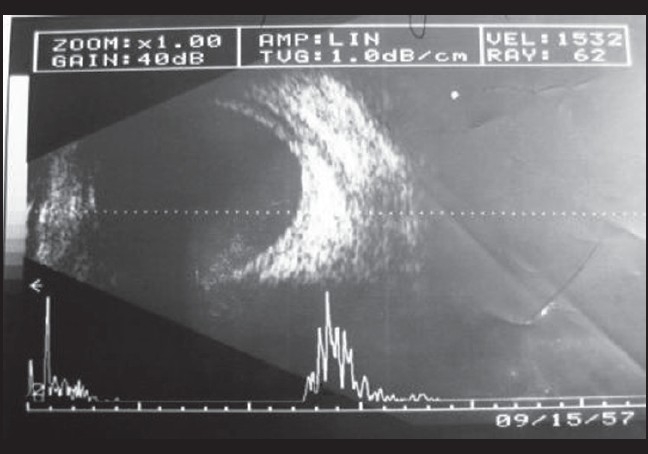
Left eye USG B-scan: choroidal detachment

On examination his best-corrected visual acuity (BCVA) was 20/40 in right eye and 20/20 in the left eye. Spectacle correction was -9.0 diopter sphere (D sph) in right eye and -9.0 D sph in the left eye and N6 without glasses. Examination revealed mild chemosis, circumcorneal congestion, conjunctival congestion, clear cornea, shallow anterior chamber more peripherally than centrally (Van Herricks Grade 1), sluggishly reacting pupil and clear lens in both eyes. IOP as measured by applanation tonometry was 20 mmHg and 22 mmHg in the right and left eyes respectively. Gonioscopy revealed 360°closed angle in both eyes. A-scan biometry of both eyes revealed axial length of 22.0 mm in both eyes. The patient underwent ultrasonography B-scan (USG B-scan) [Figs. 1 and 2] of both eyes which revealed bilateral shallow choroidal detachment, more in the superior quadrants.

**Figure 3:**
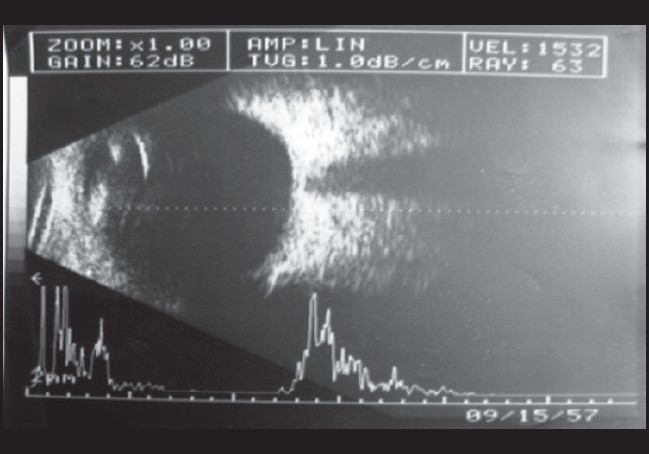
Right eye USG B-scan: resolution of choroidal detachment

**Figure 4:**
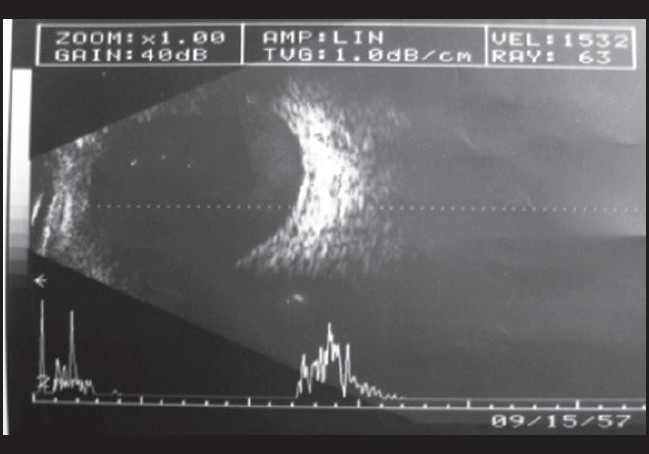
Left eye USG B-scan: resolution of choroidal detachment

With the evidence before us we came to the provisional diagnosis of bilateral secondary drug-induced angle closure glaucoma. The patient was advised eye drops timolol maleate 0.5% twice daily, dexamethosone 1% four times daily for both eyes and advised to review after seven days. On follow-up after seven days the patient was symptomatically better. His BCVA was 20/20 (N6) both eyes without correction. Anterior segment revealed no circumcornal congestion, clear cornea, deep anterior chamber (van Herricks Grade 4), normal reacting pupil and a clear lens. IOP by applanation tonometry was 10 mmHg both eyes and gonioscopy revealed 360° Grade 3 open angles in both eyes. Dilated fundus examination revealed normal optic discs with healthy neuroretinal rims in both eyes USG B-scan showed resolution of choroidal detachment [Figs. 3 and 4]. The patient was advised to avoid tablet mefenamic acid and review periodically.

## Discussion

Drug-induced angle closure glaucoma with induced myopia with choroidal detachment is a well-known entity of many sulphinamide[2]-derived medications such as acetazolamide, hydrochlorthiazide and sulphonamides, promethazine, spironolactone, isosorbide dinitrate, bromocriptine, corticosteroids, penicillamine, quinine, metronidazole, and isotretinoin. Recently, bilateral angle closure glaucoma with uveal effusions has been associated with topiramate. Of the NSAIDs only aspirin[6] has been documented as a cause of drug-induced angle closure glaucoma and myopia. Our patient developed a similar reaction after ingesting mefenamic acid for his symptoms. To the best of our knowledge there is no known published report of such a similar clinical case in the English language literature although Bialek *et al*.[7] have demonstrated mefenamic acid (and other NSAIDs)-induced permeability of retinal pigment epithelium and choroid in bovine eyes. The prompt identification of the condition helps in rapid resolution of the disease as the mainstay of treatment is to immediately stop the administration of the inciting agents as described by Chen *et al*.[8]

Desai *et al*.[9] have described a patient with topiramate-induced myopia in whom they were able to prevent angle closure glaucoma by closely monitoring the patient with suspected ocular side-effects to topiramate. They also mention that accommodative spasm, primary angle closure, posterior scleritis should be the differential diagnosis of such drug-induced myopia and secondary angle closure.

Ikeda *et al*.[10] have described ciliochoroidal effusion syndrome which encompasses ciliochoroidal effusion with ciliary body edema, shallow anterior chamber, acute angle closure glaucoma, myopic shift and lens thickening. They postulate that the swelling of the ciliary body leads to rotation of the ciliary processes around the scleral spur, obliterating the ciliary sulcus thereby pushing the lens iris diaphragm forward and shallowing the anterior chamber.

Pilocarpine was erroneously prescribed to the patient elsewhere, and in this instance, may have caused a forward movement of the lens iris diaphragm thus mechanically obstructing the trabecular outflow, raising the IOP and exacerbating the patient's symptoms. Pilocarpine should be avoided in secondary angle closure because it may lead to the development of peripheral anterior synechia and permanent damage to the trabecular meshwork.

Drug-induced secondary angle closure glaucoma is a self-limiting and transient condition which requires that it be identified at the earliest for prompt and effective treatment. Such an entity should always be kept in mind, especially in present times when drug developments are taking place at such a rapid pace. As this condition can be effectively managed conservatively, unnecessary laser or surgical intervention can be detrimental to the patient's vision and quality of life.
